# Association of Autoimmune Hyperthyroidism and Endometriosis, With an Endometrioma Rupture as a Complication: A Case Report and Review of the Literature

**DOI:** 10.1155/crog/4611384

**Published:** 2026-08-05

**Authors:** Sabahat Raees, Eric Bidinger, Casey Collier, Ligaya Marasigan

**Affiliations:** ^1^ Chicago Medical School, Rosalind Franklin University, North Chicago, Illinois, USA, rosalindfranklin.edu; ^2^ Ascension Resurrection Hospital, Chicago, Illinois, USA

## Abstract

This case report examines the association between autoimmune thyroid disease, more commonly known as Graves′ disease, and endometriosis with a focus on the complication of an endometrioma rupture. The interplay between these two conditions is of clinical interest as increased levels of estrogen are caused by immune system dysregulation. Research has depicted a positive correlation between the concentration of thyroid hormones and the size of endometriomas and/or adhesions caused by endometriosis. We report a case of an endometrioma rupture in a 31‐year‐old woman with a history of autoimmune hyperthyroidism who presented with acute abdominal pain. We are aimed at enhancing the timeliness of diagnosis and treatment by raising awareness among healthcare providers about the importance of considering endometrioma rupture in the differential diagnosis for women of childbearing age who have a history of autoimmune hyperthyroidism or endometriosis.

## 1. Introduction

Endometriomas are cystic formations filled with blood associated with endometriosis, a gynecological condition characterized by the presence of endometrial‐like tissue that exists and adheres to tissue outside the uterus. Endometriosis affects about 10% of women of reproductive age [1]. Complications of an endometrioma include spontaneous rupture, which can lead to significant morbidity and require prompt surgical intervention. The rupture of an endometrioma is a rare event, occurring in less than 3% among women of childbearing age who are known to have endometriomas [2]. The risk of rupture increases in endometriomas larger than 6 cm [2]. Endometrioma rupture should be included as a differential in women with abrupt, severe abdominal pain, regardless of previous diagnosis of endometriosis since the patient may not be aware that they have endometriosis. Patients are evaluated with diagnostic imaging including ultrasound, computed tomography (CT) scan, or MRI of the abdomen and pelvis. Transvaginal ultrasound could be considered for nondiagnostic transabdominal ultrasounds. In ultrasounds, endometriomas depict heterogeneous content, irregular contours, with hemoperitoneum if rupture has occurred, which is seen as heterogeneous fluid content [2]. Patients presenting with an acute abdomen require prompt evaluation to determine the underlying etiology. Pelvic ultrasonography (USG), particularly transvaginal ultrasonography, is the first‐line imaging modality for evaluating suspected gynecologic pathology because it readily identifies ovarian cysts, adnexal masses, free pelvic fluid, and ovarian torsion. CT is frequently obtained when the diagnosis remains uncertain or when gastrointestinal causes such as appendicitis are also being considered. CT provides valuable information regarding hemoperitoneum, complex adnexal masses, inflammatory changes, and alternative intra‐abdominal pathology. The combined use of USG and CT improves diagnostic accuracy and assists in determining the need for urgent surgical intervention in patients presenting with acute abdominal pain.

Treatment options include active surveillance, hormonal therapy, or surgical treatment and are determined by patient preference and symptoms [3]. Surgical options include conservative and definitive options, and laparoscopic surgery is preferred over laparotomy for better visualization and faster recovery [4].

The connection between autoimmune hyperthyroidism and endometriosis is documented in recent research, with some studies indicating that women with autoimmune thyroid disease are more likely to develop endometriosis [5, 6]. Elevated estrogen levels, common in both conditions, may exacerbate endometriosis and lead to complications such as the growth and rupture of endometriomas. Despite this association, endometrioma rupture is not always considered in the differential diagnosis when patients with hyperthyroidism present with acute abdominal pain, potentially delaying appropriate intervention and increasing the risk of complications.

## 2. Case Presentation

**Figure 1:**
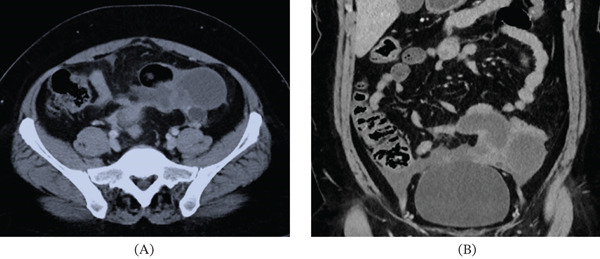
The (A) axial and (B) coronal CT findings on the patient including a large complex left adnexal cyst measured at 10.1 × 7.0 × 9.3 cm and moderate free peritoneal fluid.

A 31‐year‐old, G1P1 female with a history of autoimmune hyperthyroidism presented to the emergency department with severe right lower quadrant (RLQ) and suprapubic abdominal pain. The pain was associated with nausea that began abruptly after she was lifting weights. She had no history of prior similar symptoms. She denied vomiting and vaginal bleeding, and changes in bowel or urinary habits. Her last menses began 4 weeks prior to her presentation; however, her menses were irregular and were associated with cramping pain. She had a term pregnancy in 3 years prior to presentation, delivered via cesarean section due to labor dystocia. Her pain was rated 10/10, and her vital signs showed tachycardia but were otherwise normal. The physical exam demonstrated a patient in painful distress, with tachycardia and severe tenderness in the RLQ and LLQ, with voluntary guarding. Urinalysis, urine pregnancy, serum betaHCG, CBC, and CMP were unremarkable. CT scan of abdomen and pelvis with IV contrast displayed a large 10.1 × 7.0 × 9.3 cm complex attenuation depicting a left adnexal mass lesion with appearance consistent with a possible, large left ovarian mature cystic teratoma (Figure 1). The right adnexa was mildly loculated with surrounding peritoneal fluid. Findings demonstrated mild inflammatory intrapelvic fat stranding. The appendix abutted a portion of the right adnexal peritoneal fluid. It appeared borderline thickened suggesting perhaps minimal secondary appendicitis. Otherwise, the appendix appeared unremarkable. No additional acute CT intra‐abdominal or intrapelvic findings were identified. Due to these findings, a.

Non‐OB transvaginal ultrasound displayed normal sonographic appearance of the right ovary without evidence of ovarian torsion. The left ovary was not visualized. There was no evidence of left adnexal mass. There was moderate free fluid in the abdomen and pelvis. The appearance of the uterus and endometrium was sonographically normal.

Given the patient′s sudden onset of severe abdominal pain, the presence of a large adnexal mass, and free intraperitoneal fluid, highly suspicious for ovarian torsion and ruptured ovarian pathology. After counseling regarding the risks, benefits, and alternatives, the patient consented to diagnostic laparoscopy with possible detorsion, ovarian cystectomy, and possible oophorectomy if ovarian preservation was not feasible. General surgery consultation was also available intraoperatively should appendiceal pathology be encountered.

Diagnostic laparoscopy demonstrated copious dark hemoperitoneum with extensive blood clots throughout the abdomen and pelvis. The blood and clots were evacuated to improve visualization of the pelvic structures. Multiple ruptured multiloculated cysts involving the left ovary were identified, containing characteristic dark brown fluid consistent with endometrioma contents. Standard laparoscopic management was performed, including evacuation of hemoperitoneum, careful removal of blood clots, complete left ovarian cystectomy with excision of the endometrioma cyst wall while preserving healthy ovarian tissue, irrigation of the pelvis, and meticulous hemostasis. Bilateral hydrosalpinx and string‐like adhesions involving the anterior cul‐de‐sac consistent with endometriosis were also identified. The appendix and liver appeared grossly normal. Cytologic evaluation of the peritoneal fluid demonstrated hemosiderin‐laden macrophages, confirming rupture of an ovarian endometrioma.

## 3. Discussion

This case also highlights the importance of an organized diagnostic approach to women presenting with acute abdomen. Pelvic ultrasonography is generally the initial imaging modality because of its ability to evaluate ovarian torsion, adnexal masses, and pelvic free fluid. CT complements ultrasonography by identifying hemoperitoneum, characterizing complex adnexal lesions, and excluding gastrointestinal causes of abdominal pain. When imaging findings remain inconclusive or clinical suspicion for ovarian torsion or ruptured endometrioma persists, diagnostic laparoscopy remains the gold standard for both definitive diagnosis and treatment. Standard laparoscopic management consists of evacuation of hemoperitoneum, removal of blood clots, excision of the endometrioma cyst wall (ovarian cystectomy), preservation of normal ovarian tissue whenever feasible, and achievement of meticulous hemostasis.

It is also important to consider endometrioma rupture as a differential diagnosis in patients with autoimmune hyperthyroidism who present with acute abdominal pain. The patient, a 31‐year‐old female with a history of autoimmune hyperthyroidism, presented with severe RLQ and suprapubic pain, which was initially suspected to be related to an ovarian torsion or possibly appendicitis. However, intraoperative findings revealed multiple ruptured endometriomas and endometriosis, underscoring the need for heightened clinical suspicion for endometriosis‐related complications in similar cases.

Graves′ disease is an autoimmune thyroid disease characterized by binding of IgG antibodies to the TSH receptor. Thyroid‐stimulating immunoglobulins (TSIs) are considered pathognomonic for Graves′ disease and are positive in approximately 98% of patients. A case cohort study with 172 women detected 93% TSI positivity in endometriosis cases and only 7.9% positivity in the control group. A significant association was made between TSI levels and endometriosis prevalence [7]. It is speculated that endometrial tissue could produce extrathyroidal hormone production in response to TSH. Additionally, non‐uterine endometrial implants express TSH receptors and can induce TSI formation. In the study, the mean TSI levels were found to be higher in the endometriosis group, although the difference was not significant. Larger sample sizes and more focused evaluations of women with endometriomas are required to determine the strength of association between endometriosis and autoimmune hyperthyroidism. This case study presents the association between hyperthyroidism and endometriomas and warrants further assessment.

Furthermore, endometrioma growth is estrogen‐dependent, with local estrogen production contributing to its enlargement. The risk of spontaneous rupture increases with the size of the endometrioma. Differential expression of the estrogen receptor beta gene (ESR2) has been confirmed through microarray and immunohistochemistry in patients with endometriosis [8]. A research study conducted in Poland involving 375 patients with Graves′ disease revealed a statistically significant frequency of this ESR2 gene, depicting the association of Graves′ disease to endometriosis [9]. The increased function of ESR2 likely serves as a common link between Graves′ disease and endometriosis, possibly contributing to endometrioma growth and increasing the risk of rupture.

The association of autoimmune hyperthyroidism and endometriosis is not common knowledge among physicians. Providers should be aware that patients with autoimmune hyperthyroidism are at an increased risk for endometriosis and related complications. Recognizing these associations early can lead to more timely and appropriate management, reducing the likelihood of severe outcomes such as cyst rupture, extensive adhesions, and chronic pain.

## 4. Conclusion

Endometrioma rupture should be considered in the differential diagnosis of reproductive‐aged women presenting with acute abdominal pain who have autoimmune hyperthyroid disease, even in the absence of a formal endometriosis diagnosis. Prompt evaluation with ultrasonography and, when indicated, CT can facilitate early diagnosis, whereas laparoscopic surgery remains the standard approach for definitive diagnosis and treatment. Larger sample sizes and more focused evaluations of women with endometriosis are necessary to further assess the association between endometriosis and Graves′ disease.

Although additional studies are needed to further clarify the relationship between Graves′ disease and endometriosis, this case contributes to the growing body of evidence suggesting an association between autoimmune hyperthyroidism and endometriosis. Increased awareness among healthcare providers may promote earlier recognition of endometriosis‐related complications in patients with autoimmune hyperthyroidism, reduce diagnostic delays, and improve patient outcomes.

## Author Contributions

**Sabahat Raees** made significant contributions to the literature review and drafting of the article; **Eric Bidinger**, MD, **Casey Collier**, MD, and **Ligaya Marasigan**, MD, critically revised the article for essential intellectual content; Dr. **Ligaya Marasigan** granted final approval for the article′s publication; Dr. **Casey Collier** supplied the CT abdomen images and data.

## Funding

No funding was received for this manuscript.

## Disclosure

All authors accept responsibility for all aspects of the work, ensuring that any questions regarding the accuracy or integrity of the work are thoroughly investigated and addressed.

## Ethics Statement

This case report received ethical approval from our institution. The patient completed the written consent form upon admission to the emergency department.

## Conflicts of Interest

The authors declare no conflicts of interest.

## Data Availability

Data sharing is not applicable to this article as no datasets were generated or analyzed during the current study.
